# Supplementary material for: Anatomical and functional investigation of the marmoset default mode network
Source: Nat Commun. 2019 Apr 29;10:1975. doi: 10.1038/s41467-019-09813-7 (PMC6488610; doi:10.1038/s41467-019-09813-7)
Supplement: Supplementary file 1 — Supplementary Information [file 41467_2019_9813_MOESM1_ESM.pdf]

Title: Anatomical and functional investigation of the marmoset default mode network

Authors: Liu et al.

Supplementary Figures

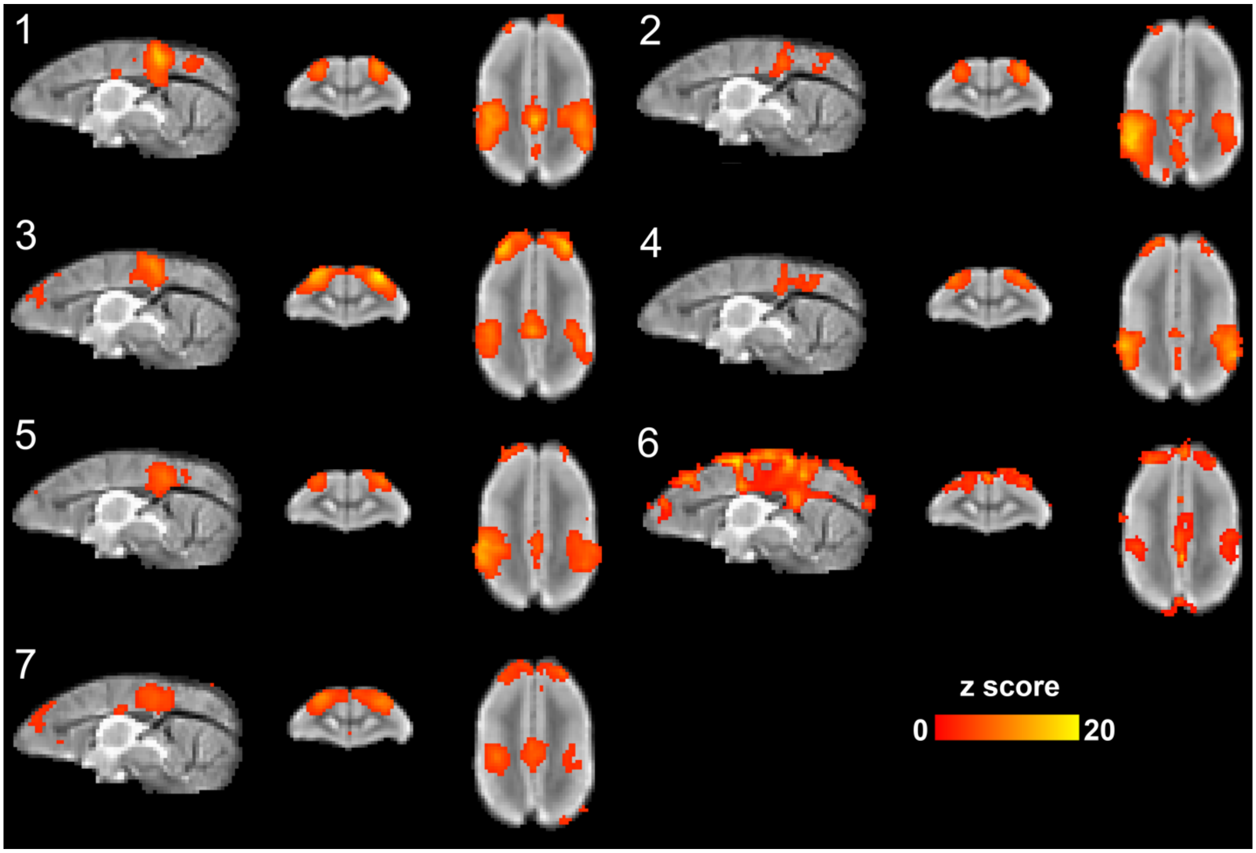

Supplementary Figure 1. The subject-specific ICA DMN components for each marmoset. ICA components are thresholded at a level of 0.5 using a mixture model and an alternative hypothesis testing approach (default of the FSL-Melodic).

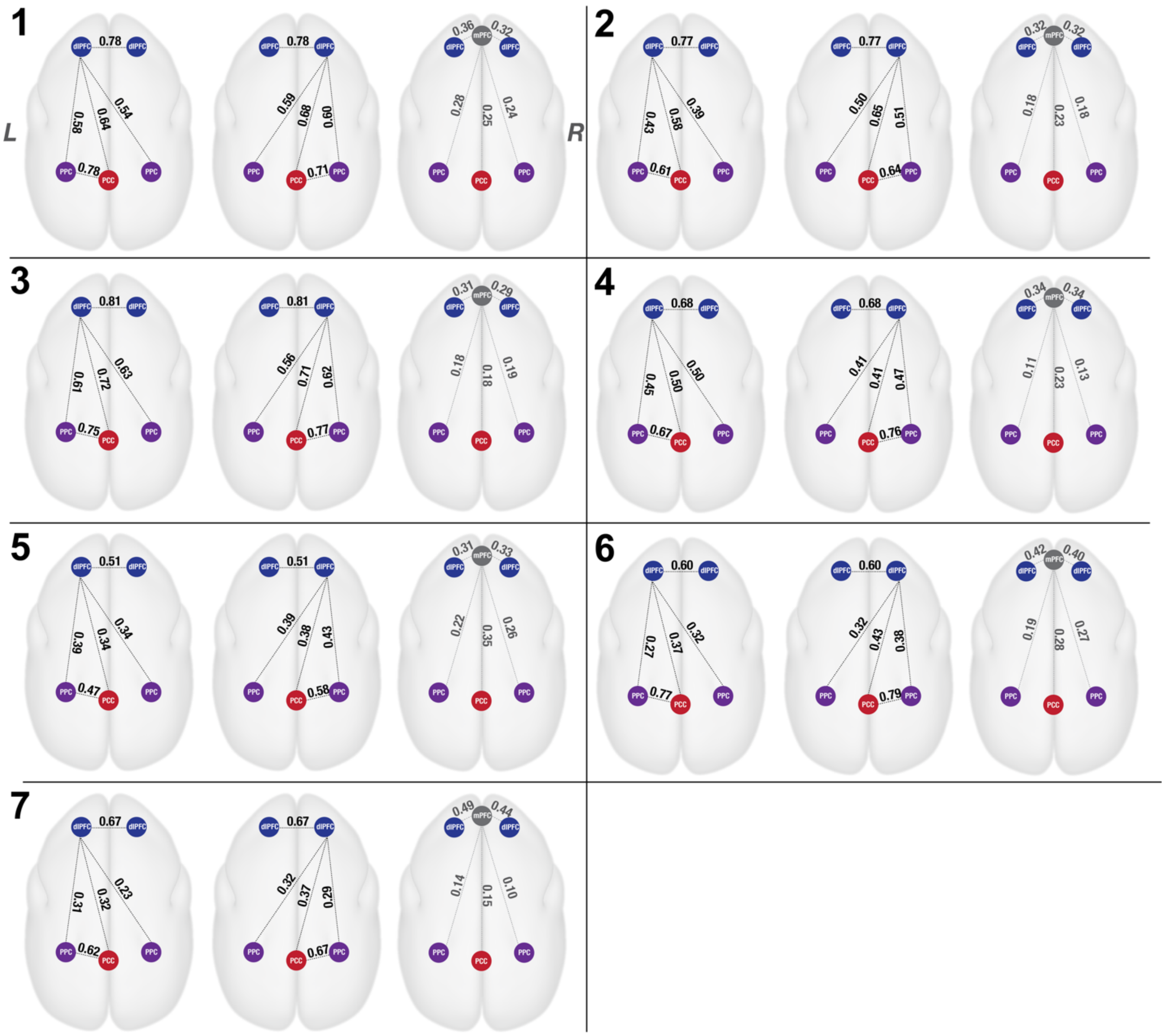

Supplementary Figure 2. Resting-state functional connectivity between the different DMN regions for each marmoset.

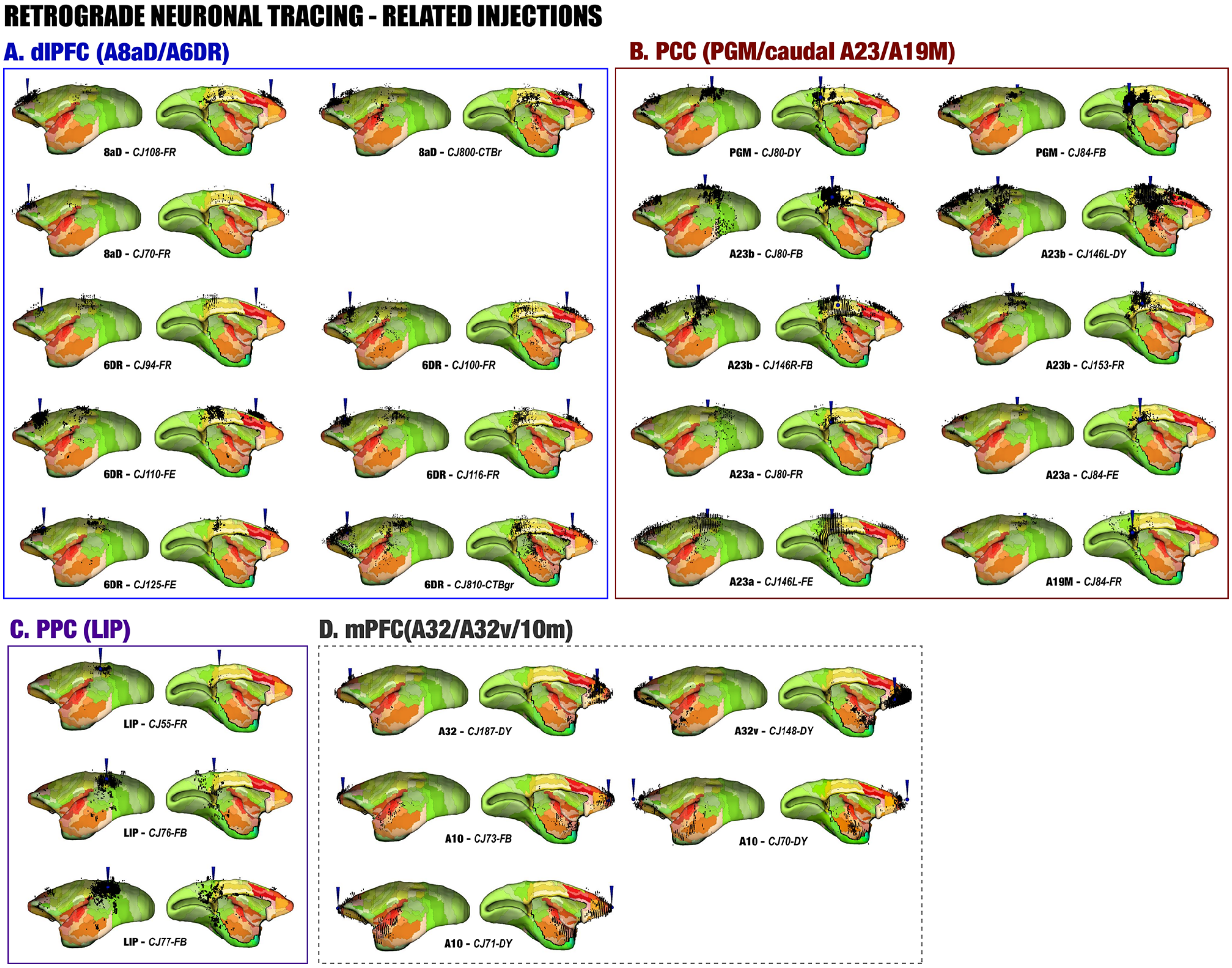

Supplementary Figure 3. More neuronal tracing examples of related regions from the Monash Marmoset Brain Architecture Project [14].

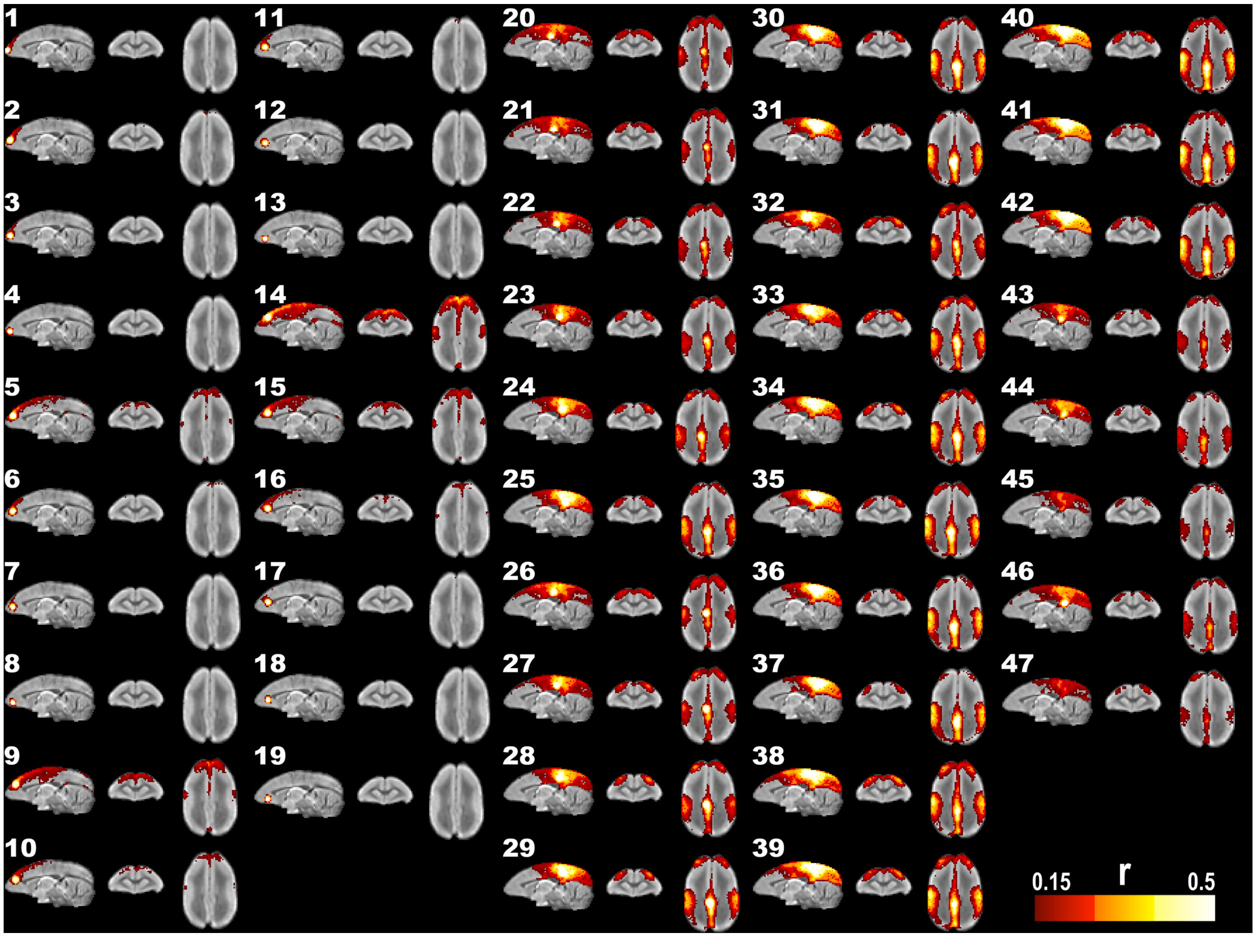

Supplementary Figure 4. Correlation maps of all seeds from the exploratory seed-based analysis with correlation threshold r > 0.15.

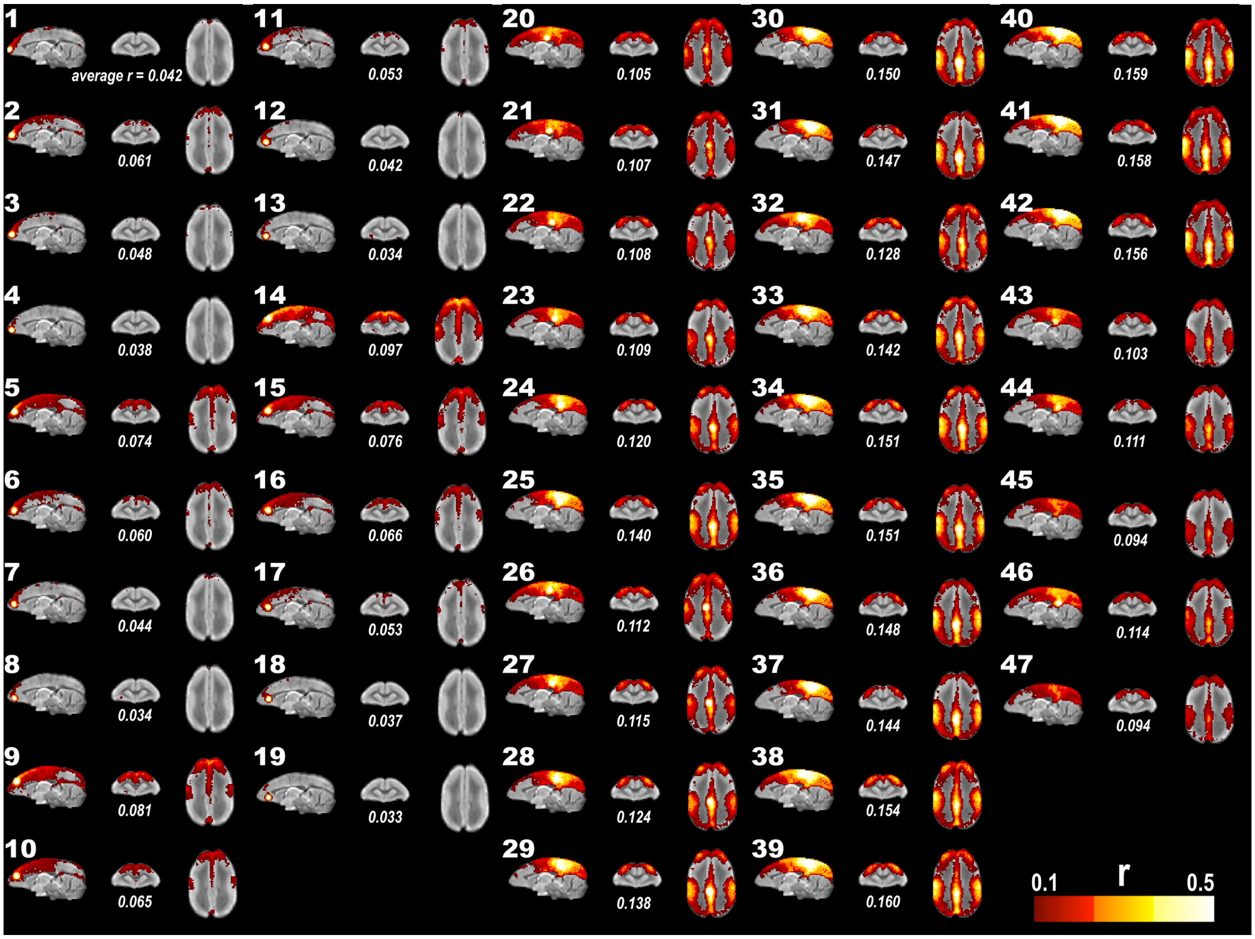

Supplementary Figure 5. Correlation maps of all seeds from the exploratory seed-based analysis with correlation threshold r > 0.1. Note that the r = 0.1 is a very low threshold, as for many seeds, their average positive correlations over the whole cortex is more than 0.1**.** The average positive correlation over the whole cortex is also label for each seed at the bottom of their coronal slices.
